# Variation in genetic admixture and population structure among Latinos: the Los Angeles Latino eye study (LALES)

**DOI:** 10.1186/1471-2156-10-71

**Published:** 2009-11-10

**Authors:** Corina J Shtir, Paul Marjoram, Stanley Azen, David V Conti, Loic Le Marchand, Christopher A Haiman, Rohit Varma

**Affiliations:** 1 Department of Human Genetics and Neuroscience, University of California - Los Angeles, Los Angeles, CA 90095-708822, USA; 2 Department of Preventive Medicine, Division of Biostatistics, Keck School of Medicine, University of Southern California, Los Angeles, CA 90033, USA; 3 Department of Environmental Health, Keck School of Medicine, University of Southern California, Los Angeles, CA 90033, USA; 4 Epidemiology Program, Cancer Research Center, University of Hawaii, Honolulu, Hawaii, USA; 5 Department of Ophthalmology, Keck School of Medicine, University of Southern California, Los Angeles, CA 90033, USA; 6 Doheny Eye Institute, Keck School of Medicine, University of Southern California, Los Angeles, CA 90033, USA

## Abstract

**Background:**

Population structure and admixture have strong confounding effects on genetic association studies. Discordant frequencies for age-related macular degeneration (AMD) risk alleles and for AMD incidence and prevalence rates are reported across different ethnic groups. We examined the genomic ancestry characterizing 538 Latinos drawn from the Los Angeles Latino Eye Study [LALES] as part of an ongoing AMD-association study. To help assess the degree of Native American ancestry inherited by Latino populations we sampled 25 Mayans and 5 Mexican Indians collected through Coriell's Institute. Levels of European, Asian, and African descent in Latinos were inferred through the USC Multiethnic Panel (USC MEP), formed from a sample from the Multiethnic Cohort (MEC) study, the Yoruba African samples from HapMap II, the Singapore Chinese Health Study, and a prospective cohort from Shanghai, China. A total of 233 ancestry informative markers were genotyped for 538 LALES Latinos, 30 Native Americans, and 355 USC MEP individuals (African Americans, Japanese, Chinese, European Americans, Latinos, and Native Hawaiians). Sensitivity of ancestry estimates to relative sample size was considered.

**Results:**

We detected strong evidence for recent population admixture in LALES Latinos. Gradients of increasing Native American background and of correspondingly decreasing European ancestry were observed as a function of birth origin from North to South. The strongest excess of homozygosity, a reflection of recent population admixture, was observed in non-US born Latinos that recently populated the US. A set of 42 SNPs especially informative for distinguishing between Native Americans and Europeans were identified.

**Conclusion:**

These findings reflect the historic migration patterns of Native Americans and suggest that while the 'Latino' label is used to categorize the entire population, there exists a strong degree of heterogeneity within that population, and that it will be important to assess this heterogeneity within future association studies on Latino populations. Our study raises awareness of the diversity within "Latinos" and the necessity to assess appropriate risk and treatment management.

## Background

Recent years have seen great advances in discovering genetic variants associated with the biogenesis and progression of a variety of complex diseases (e.g., [1-8]). Despite the relative success of mapping susceptible loci, we are still faced with a frequent lack of replication across different populations. One possible cause is our relatively poor understanding of the degree of genetic diversity *between *populations. Besides the variation in genetic make-up across ethnicities, we often observe a wide range in incidence and prevalence rates across populations, for any given disease; it is likely that this range is largely due to that variation.

On the other hand, population substructure may inflate positive associations and cause hidden confounding effects due to an underlying difference in the distribution of ancestry between cases and controls [9-19]. If a particular ancestral group has relatively lower disease prevalence rates, this will result in an under-representation of that subgroup in cases versus controls. Loci with dissimilar allele frequencies across populations may induce spurious associations with phenotype. For example, the *CY3A4-V *gene variant and prostate cancer are reported to be substantially less common among European American than African American (*AA*) men; Kittles *et al*. studied 688 *AA*s and found that a strongly significant association at *CYP3A4-V *for prostate cancer became a non-significant signal after including ten ancestry informative markers (AIMs) [19]. Several discrepancies in both disease prevalence rates and genetic susceptibility loci have been confirmed in Latino studies. For instance, Salari *et al*. [20] found a higher level of European ancestry among Mexican Americans to be strongly associated with increased asthma severity, while a higher proportion of Native American ancestry was protective. Also, Choudhry *et al*. (2006) observed a significant difference in allele frequencies between asthma cases and controls (P = 0.0002) in Puerto Ricans, but not in Mexicans.

As Latinos form the largest minority ethnic group in the US, with close to 100 million individuals projected by 2050 [21], a growing number of genome-wide association studies will involve that population. It is therefore essential to understand the specifics of genetic structure within Latino populations, and to design association studies with reference to that structure. Thus, we examine the ancestral landscape of Latinos ascertained through the Los Angeles Latino Eye Study (LALES), the largest visual impairment epidemiologic cohort of Latinos in the US [22]. As such, this cohort represents a unique opportunity to better decipher the demographics of Latinos.

The LALES study is a population-based cohort composed of 6,357 Latinos residing in 6 census tracts of the Los Angeles County, who originated mainly in the US, Mexico, Guatemala, or El Salvador. Preliminary evidence suggests that there are differences for risk of AMD between various populations [23-32]. While prevalence rates for early AMD among Latinos are similar to those found in Caucasians [9.4% LALES vs. 7.2% Blue Mountains Eye Study (BMES) vs. 15.6% Beaver Dam] and in individuals of African descent (12.6% BES) [27,29,31,32], incidence data indicates that only 1.5% of early AMD cases advance into late AMD in Latinos, while 3.4% of cases progress in Caucasian cohorts. Despite the growing evidence for the role of complement pathway in development of AMD, discordant frequencies for a series of AMD risk alleles have been reported between different ethnic groups [24,31-35].

The difficulty in defining Latino admixture rests in our relatively poor historical understanding of the demographic events that converged into shaping the modern Latinos from the source populations of the Americas, Europe, Asia and Africa. However, the history of any population is written in its genetic make-up, and that version is forgotten much more slowly than any language-based version of the same history. While a number of studies defined the admixed nature of Latinos to be mostly composed of Native American and European descent [20,36-39], there is a considerable degree of heterogeneity within Native Americans. Wang *et al*. examined genetic diversity in 29 Native American populations from North, Central, and South America, and compared them to Siberian populations [40]. They depicted gradients of decrease in both genetic diversity and similarity to immigrant Siberians as a function of geographic distance from the Bering Strait. Unfortunately, the relative paucity of available genome-wide data for the Native American populations has made even the genetic data hard to interpret. Consequently, in addition to the data inherent in the LALES study, we have also generated genotype data for a number of Native American individuals.

Previous studies identified ancestry informative marker (AIM) polymorphisms that exhibit large differences in allele frequencies across populations of European, Asian, and African descent, and therefore confer increased power for detecting levels of population stratification [38,41-44]. A series of projects have since followed, describing the effects these ancestries have on numerous genetic risk factors [18,45-55]. However, such AIMs are liable to be less powerful when describing the ethnicity of Latinos. For example, Mexican Americans contain a rather small percentage of African heritages and are mostly composed of a mixture of European and Native American ancestry [20,36,47,50-52]. The historical focus on the HapMap has meant that a clear and comprehensive description of genetic admixture among American Latinos has been lacking, and has only recently started to emerge [20,37,38,56]. Our analysis uses AIMs genotyped for 6 population samples: (1) LALES Latinos, (2) Native Americans selected through Coriell's institute for medical research laboratory http://ccr.coriell.org, (3) Yoruba Africans (YRI) from the HapMap II database, (4) Asian, African and European descent individuals from the USC Multiethnic Panel (USC MEP), consisting of samples from the Multiethnic Cohort (MEC) [57,58], and (5-6) two additional Chinese cohorts [59,60]. We use this set of marker data to infer the important demographic characteristics of Latinos. This will enable investigators to increase the power of future association studies based on Latino populations.

## Results

### LALES demographics

A total of 500 out of 538 genotyped subjects were included in the final analysis after a sample call rate test was performed at the 0.80 level. Age, gender, and self-reported geographic birthplace distributions for the 500 LALES subjects are given in Table 1. Recent Latino-based population studies reported various ancestry estimates between Puerto Ricans and Mexican Americans [20,36,39,61]. Overall, LALES birth locations were dispersed as 68.4% Mexico, 18.2% USA, 5.4% El Salvador, 3.4% Guatemala, and 4.6% from other places. There is little difference between cases and controls in this respect, as would be expected given that the inclusion criteria for cases and controls in the original LALES cohort (n = 6357) study design required a matched frequency for birthplace location.

**Table 1:** LALES sample demographics

LALES Demographics		Cases	Controls
**Age Average (S.D.)**	All (n = 500)	60.34 (11.37)	60.04 (11.54)
	Males (n = 227)	59.62 (11.37)	60.74 (11.55)
	Females (n = 273)	61.12 (12.40)	59.61 (11.54)

**Birthplace %**	Mexico	68.0	68.8
	USA	18.0	18.4
	EL Salvador	5.6	5.2
	Guatemala	3.6	3.2
	Other	4.8	4.4

Note.

S.D. = Standard Deviation

n = Number

Other = Other birthplace locations

### Estimation of LALES Population Structure and Admixture

**Figure 1:**
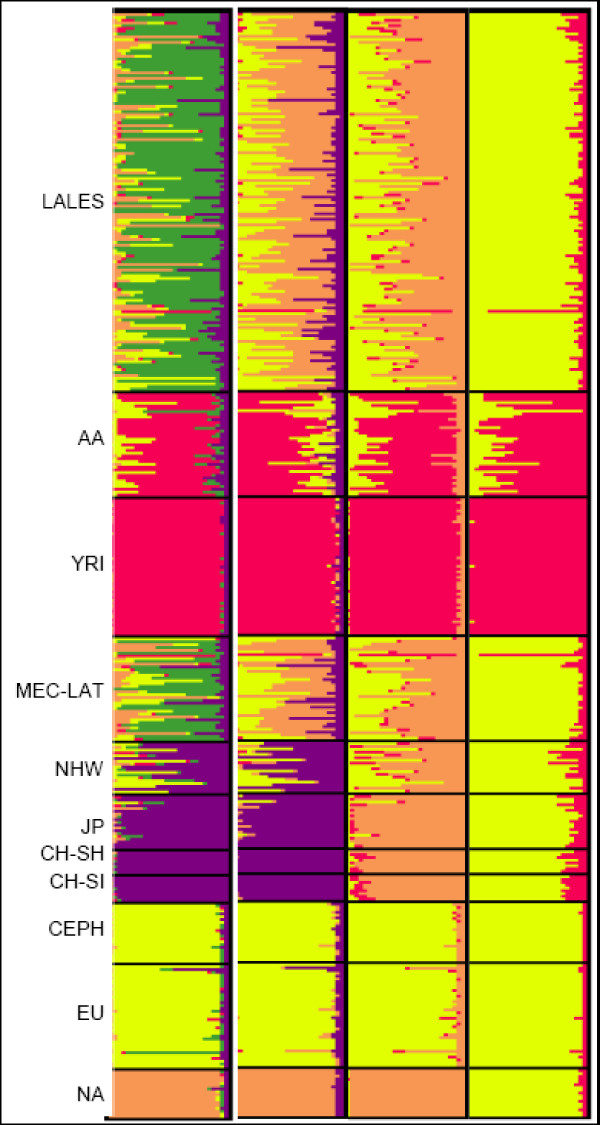
**Individual ancestry proportions for the LALES, Multiethnic Cohort, and Native American sampled populations**. Population Number Code: *AA *- MEC African Americans; MEC-LAT - MEC Latinos; JP - MEC Japanese (*AS*); NH - MEC Native Hawaiians; CH-Sh - China Shanghai (*AS*); CH-Si - China Singapore (*AS*); CEPH - CEPH (*EU*); MEC-EU - MEC Europeans (*EU*); LALES - LALES Latinos; *NA *- Native American; YRI - Yoruba Africans

**Figure 2:**
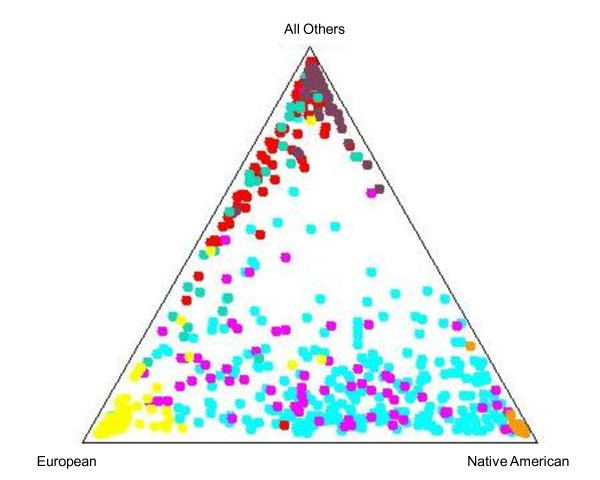
**Cluster ancestry distribution for the LALES, Multiethnic Panel, and Native American samples**. Each individual is positioned proportional to his/her ancestral similarity to each the three reference groups. Individuals placed at a particular corner are completely assigned to the corresponding population, whereas those in the centroid area are equidistant from each of the three group lineages. LALES Latinos - turquoise; MEC Latinos - pink; African - red; Asian - purple; European - yellow; Native Hawaiian - green; Native American - orange.

Population structure for the LALES, YRI, USC MEP, and *NA *samples for each of the *K *= {2, ..., 5} cluster models are illustrated in Figure 1. Reported results represent an average from 3 different runs, all of which gave consistent results, reflecting proper MCMC convergence. For STRUCTURE analysis estimates see Additional file 1, Table S1; the log likelihood of the data, *ln*Pr(X|*K*), and the corresponding allele frequency difference measure F_K _are summarized for each *K *= {2, ..., 5}. Previous studies suggest that Latinos are a mixture of three main source populations (Native American, European, and Asian), with rather little African descent [20,36,40,47,61]. For this reason, we focus on the modeling results of *K *= 4 for which the second largest likelihood [*ln*Pr(X|*K *= 4) = -116312.20] where the average LALES Latino admixture is partitioned as 45.2 - 54.3% Native American, 32.1 - 40.1% European, 9.7 - 11.5% Asian, and 4.0 - 5.2% African-American (Tables 2 and 3). We estimated Latino admixture proportions from the inclusion of LALES controls only (n = 250). Nucleotide distance dispersions of individual ancestry vectors for K = 4 are plotted in Figure 2, where each individual is mapped on the triangular coordinates between Native American, European and 'Other' ethnicities

**Table 2:** Estimation of ancestry proportions for the LALES, MEC/Chinese/CEPH, and Native American populations

Population	*AF*	*EU*	*AS*	*NA*
MEC African American	0.721	0.167	0.066	0.047
MEC Native Hawaiian	0.031	0.329	0.591	0.049
MEC Japanese	0.012	0.034	0.892	0.061
Chinese - Shanghai	0.013	0.018	0.916	0.052
Chinese - Singapore	0.014	0.02	0.934	0.032
MEC European	0.009	0.939	0.029	0.022
CEPH	0.015	0.891	0.056	0.038
Native American	0.005	0.013	0.032	0.949
MEC Latinos	0.059	0.453	0.116	0.373
LALES Latinos	0.049	0.401	0.098	0.452

Note.

Estimates for the LALES Latino population are based on the LALES controls.

European - *EU*; African - *AF*; Asian - *AS*; Native American - *NA*.

**Table 3:** Estimation of ancestry proportions for the LALES Latinos based on 111 SNPs from two admixture models: (1) African American, European, Native American, and Asian source populations, and (2) Yoruba African, European, Native American, and Asian source populations.

Source Populations	LALES Ancestry Estimates	*NA*	*EU*	*AF*	*AS*
**AA, AS, NA, EU**	All	0.480	0.352	0.052	0.115
	El Salvador/Guatemala	0.484	0.259	0.095	0.163
	Mexico	0.444	0.369	0.050	0.136
	USA	0.320	0.458	0.051	0.171

**YRI, AS, NA, EU**	All	0.543	0.321	0.040	0.097
	El Salvador/Guatemala	0.596	0.230	0.084	0.091
	Mexico	0.539	0.332	0.034	0.095
	USA	0.453	0.394	0.025	0.128

Note.

Estimates for the LALES Latino populations are based on the LALES controls.

African American - *AA*; African - *AF*; Asian - *AS*; European - *EU*;

Native American - *NA*; Yoruba African - *YRI*.

In comparison to LALES Latinos, those ascertained through the MEC cohort show a stronger relatedness to Europeans (~40.1% vs. ~45.3%) with correspondingly lower Native American ancestry (~45.2% vs. ~37.3%) (Table 2). This discrepancy is likely to be a consequence of differentiation in selection of individuals for the two cohorts from the different birth places. Roughly 18% of the LALES Latinos were born within the US and 68% within Mexico, with smaller proportions born in Guatemala and El Salvador (Table 1). For the MEC sample these proportions are somewhat different, with 47% of Latinos born in the US, 34% in Mexico, 10% in Central/South American, and 4% in Cuba. Three MEC Latino individuals were of unknown birth origin.

When we split the data by birth origin (i.e. US vs. Mexico vs. Central/South America or El Salvador/Guatemala), even though there are some differences in *EU *and *NA *proportions between MEC and LALES Latinos, we detect in both cohorts a gradient of linear increase in *NA *ancestry from North (US) to South (El Salvador for LALES or South America for MEC) with a corresponding decrease in European descent (Table 4).

**Table 4:** Estimation of ancestry proportions for the LALES and MEC Latinos by birthplace location

Latinos	Birth Region	*NA*	*EU*	*AS*	*AF*
LALES	El Salvador + Guatemala	0.52	0.30	0.12	0.07
	Mexico	0.49	0.37	0.11	0.04
	USA	0.42	0.42	0.12	0.04
	Other	0.35	0.42	0.10	0.14

MEC	Central/South America	0.51	0.37	0.06	0.05
	Mexico	0.39	0.39	0.08	0.13
	USA	0.35	0.47	0.04	0.14

Note.

LALES = Los Angeles Latino Eye Study

MEC = Multi Ethnic Cohort

*EU *- European; *AF *- African; *AS *- Asian; *NA *- Native American

Moreover, individual *NA *and *EU *ancestry distributions between Salvadorans/Guatemalans and the rest of the LALES cohort were significantly different (Wilcoxon signed P-values = 0.012 and 0.009, respectively). Since relatively few individuals were born in El Salvador and Guatemala, we included both LALES cases and controls for the computation of Wilcoxon tests. We note however that separate analyses of LALES cases or controls gave very similar ancestry estimates (Additional file 1, Table S2), resulting in non-significant differences (P-values > 0.5) for any of the *NA*, *EU*, *AF*, or *AS *proportions.

All 223 MEC markers were selected from the admixture map panel developed by Smith *et *al. (2004). The authors estimate these markers (3,011) to be optimal for distinguishing European, West African, Amerindian, and East Asian mixtures. Recent studies have identified extensive heterogeneity across African populations [62,63]; the STRUCTURE analysis depicted 14 ancestral clusters across Africa. This issue is also relevant for Native American populations; Wang *et *al. [40] and Tishkoff *et *al. [62] both report high variation among Native Americans. For this reason we sought to include Native Americans that co-inhibit the same regions as most of our LALES cohort. We note that the MEC study ascertained African Americans rather than Yoruban Africans (*YRI*). To compare Latino ancestry estimates derived from *AA*s vs. *YRI*s we performed parallel Structure analyses for a subset of 111 AIMs identified in the *YRI *HapMap II database; this resulted in an overall increase in NA ancestry of ~6% (54.3% vs. 48.0%) and a corresponding decrease in *EU *origin of ~3% (35.2% vs. 32.1%) when *YRI*s rather than *AA*s were set as founders (Table 3). However, some degree in variation will result from using the smaller set of 111. To examine the potential extent of this variation we selected random samples of 111 SNPs from the total of 176 SNPs that passed the call rate threshold of 0.98. The average ancestry estimates across LALES Latinos ranged from 42.2% to 51.4% *NA *and from 32.1% to 37.9% *EU *(Additional file 1, Table S3). However, regardless of the admixture model or the set of markers analyzed, the North to South trend among Latino populations for NA and EU mixtures remains the consistent; lowest NA heritage within US born Latinos, and highest within El-Salvador/Guatemala.

While, for ease of interpretation we focus our results on the assumption of four source populations, the strongest log-likelihood was obtained at *K *= 5 for both the *AA *and *YRI *based analyses [*ln*Pr(X|*K *= 5) = -116186.10 and -80348.2, respectively vs. *ln*Pr(X|*K *= 4) = -116312.2 and - 81256.7, respectively]. The 5^th ^cluster explains in both analyses approximately 63.0% of LALES and 47.6% of MEC Latino ancestry, though this substructure is found in none of the founder populations (Figure 1; Additional file 1, Table S4).

### Selection of markers informative for distinguishing between Native American and European ethnicity

It would clearly be useful to determine a set of SNPs that might be helpful in untangling admixture in Latinos, but the HapMap data contains no Native American individuals. With this in mind, Table S5 (see Additional file 1) summarizes the chromosomal positions and allele frequencies of 42 SNPs for which we detected at least 30% difference in allele frequencies (*δ *> 0.3) between *NA *and *EU *populations. This set of markers offers an addition to the previously reported Latino population admixture map markers provided by Price *et *al. (2007) [37].

### Tests for population structure and recent admixture

The HWE test was used as a means of detecting population structure and/or recent admixture. While none of the 176 AIMs failed HWE, the overall distribution of genotype homozygosity showed a greater shift to the right (higher homozygosity) in the LALES Latinos than in any of the founder populations (Additional file 2, Figure S1). This tendency is reduced in the MEC Latinos. Additional Figure S2 (see Additional file 3) reveals a potential explanation for this. We examined the distribution of homozygosity within the LALES population for those born within vs. outside the US. Given that the MEC Latino population contains a larger proportion of individuals born within the US, a smaller signature of increased homozygosity might be expected.

Finally, from a total of 15,931 pair-wise SNP combinations we obtained a subset of 15,163 pairs formed by SNPs positioned on different chromosomes; 10.0% of the unlinked pairs were significantly associated in the LALES cohort compared to 6.7% in MEC Latinos. These results point towards evidence for recent population admixture in Latinos that have recently populated the US, as they compose ~82% of the LALES vs. 50% of the MEC cohort.

### Effect of Sample Size on Admixture Estimation

We used two sampling techniques to explore the effect of relative sample size on inferred ancestry. In a first approach, we sub-sampled the LALES cohort to produce a sample of size 70, broadly consistent with the other samples in our data. Despite the wide variation of estimated *NA *and *EU *admixture proportions within LALES individuals, this approach typically resulted in estimates broadly similar to those resulting from the initial dataset analysis (Additional file 1, Table S6). Estimated *NA *and *EU *ancestries had a mean (s.d.) over 100 sampled datasets of 45.0% (2.0%) and 42.0% (2.0%), respectively, compared to original estimates of 45.2% and 40.1%. Using a second bootstrapping approach (sampling with replacement) we increased smaller datasets to 250 individuals each, matching the size of the LALES control set. We report average ancestry estimates over 100 samples (Additional file 1, Table S6; Additional file 4, Figure S3). Mean *EU *ancestry in LALES Latinos increased to 44.3% (s.d. = 0.6%), with a correspondingly lower *NA *percentage (42.2% (0.7%)). While this outcome is only suggestive, it does seem that a sample size of 70 individuals per ethnic group is sufficient to obtain reliable estimates, at least in the present context. However, if there is a perceived need to increase the size of smaller samples by using boot-strapping, somewhat altered estimates of admixture proportions may result.

## Discussion

Association studies of recently admixed populations may produce spurious allelic associations for markers that are in linkage disequilibrium with a causal gene, a reason for replication failures in other populations [9,16,18,64]. It is therefore necessary to first assess the extent of admixture when designing association studies that involve populations such as Latinos. The degree of genetic variation within 'Latino' populations is not well understood, so in this paper we evaluated admixture in Latinos ascertained through the Los Angeles Latino Eye Study, the most comprehensive eye disease study in the US. Our paper raises awareness of the diversity within "Latinos" themselves and provides a resource for future invasive examination of ancestry-specific AMD mechanisms or other related biological pathways. A distinctive characteristic of the LALES study is the ascertainment of Latinos from different geographic regions, an aspect that allowed us to better characterize the extent of Native American and European variation.

Depending on the details of which SNPs were incorporated in our analysis and, correspondingly, which African populations were used as a reference, the LALES Latinos were estimated to inherit in the region of 50% NA and 40% EU ancestry. This reflects the importance of structure within reference populations, such as the Africans here, as well. However, whichever set of Africans was used as a reference, we observed a consistent trend for Native American ancestry to increase on a north (lowest) to south (highest) gradient within the Americans. It is also important to note that our study focused on using *K *= 4 clusters (*AF*, *AS*, *EU*, and *NA*) in the STRUCTURE analysis, whereas earlier studies used *K *= 3 (*AF*, *EU*, and *NA*) [20,38]. When we replicate the approach of Salari *et *al. (2005) and of Collins *et *al. (2004), by excluding Asians and running an analysis with *K = 3 *we recover broadly the same estimated ancestry proportions in both Mexican LALES Latinos (53.4% *NA *and 40.3% *EU*) and the overall LALES cohort (49.3% *NA *and 41.1% *EU)*.

Increased homozygosity is a commonly-used signature for admixture. We observe elevated levels of homozygosity in Latinos. The increase is higher in the LALES Latinos than in those from the MEC cohort, an indicator of more recent population admixture among Latinos that have migrated recently to the US. Indeed, when we compared US with non-US born LALES Latinos, we observed an increase in the level of homozygosity in the latter. Another indicator of recent admixture and/or population structure is the degree of allelic association between markers positioned on different chromosomes. 10% vs. 6.7% of unlinked locus pairs were associated in LALES vs. MEC Latinos, an additional confirmation of heterogeneity within Latinos. Finally, in an attempt to aid the design of future studies involving Latinos, we reported a set of SNPs with high differences in allele frequencies between Native Americans and Europeans.

The issue of whether the results from a STRUCTURE analysis are affected by discrepancies between sample sizes across ethnic groups is not typically addressed. Our results suggest two things. First, unequal sample sizes do not appear to bias estimates of ancestry, at least in the context of the present paper. Second, they support the belief that sample sizes of 25 or great are typically sufficient to give meaningful estimates of ancestry. Finally, when we tried another common strategy, inflating sample sizes by boot-strapping, ancestry estimates did appear to change from those found in the original sample. While these results are clearly only suggestive, they do imply that caution should be exercised before employing such an approach. However, we also note that the standard deviation of the estimates appears to decrease as sample-size increases, as would be expected. The relative merits in the trade-off between the apparent change in ancestry estimates in the boot-strapped samples and the decrease in standard deviation of those estimates, remains to be assessed in future studies.

## Conclusion

In summary, we found strong evidence for recent population admixture in Latinos ascertained through the LALES cohort. By specifically incorporating, and in some cases collecting genotype data for each of the likely source populations, we were able to identify the ethnicity related to each component of the Latino genetic make-up. The highest ancestral component was Native American, with gradients of increasing *NA *ancestry as a function of birth origin from North to South (US, Mexico, Guatemala, El Salvador). These findings reflect the historic migration patterns of the *NA *population and suggest that while the 'Latino' label is used to categorize the entire population, there exists a strong degree of heterogeneity within that population, and that it will be imperative to assess this heterogeneity and control for it within future association studies using Latino populations.

## Methods

### Selection of ancestry informative markers (AIMs)

We used a set of 233 AIMs, dispersed throughout the genome, and chosen from a set of high-density admixture map markers described in Smith *et *al. [65]. These SNPs exhibit a substantial difference in allele frequencies across ethnicities [66]. In addition, AIMs are specifically chosen to lack linkage with any known human disease candidate. These SNPs had been previously genotyped among the USC MEP. Given the existence of this data, and our desire to incorporate it within our study, we ourselves genotyped the LALES sample and the *NA *collection of individuals at the same set of AIMs.

### Study Subjects

Six datasets were compiled for the estimation of Latino ancestry for the ongoing ocular disease study of the LALES cohort: LALES, *NA, YRI*, and a multiethnic panel comprised of subjects from the MEC and two Chinese cohorts. We genotyped two distinct datasets for the same set of AIMs described above: (1) 538 LALES subjects and (2) 30 Native Americans. A brief description of the LALES, NA, and MEC datasets is provided below. Ninety *YRI *samples from the HapMap II project were incorporated in the population admixture models.

### LALES Subjects

538 LALES participants (268 cases: 268 controls) with an average age (s.d.) of 56.7 (11.2) years were genotyped for this study (Table 1). All LALES cases were diagnosed with early AMD through the detection of bilateral, intermediate to large soft drusen deposits. Controls lacked drusen in either eye and were matched with cases based on age and birthplace location. Details of the LALES cohort design are described elsewhere [22,67,68]. All procedures followed the Declaration of Helsinki for research involving human subjects. The Los Angeles County/University of Southern California Medical Center Institutional Review Board approved the project, and informed consent was obtained from all participants.

### Native American Subjects

In order to establish a reference set for the *NA *lineage in Latinos, we genotyped 25 Mayan Amerindian and 5 Mexican Indian DNA samples from Coriell's human population repository collection http://ccr.coriell.org/. The Mayan samples were specifically chosen because they represent ancient Native American civilizations that lived before the arrival of Europeans in what nowadays are eastern and southern Mexico, El Salvador, Guatemala, Belize, and Honduras. Since the dispersion of geographic regions for the LALES cohort covers Mexico and most of Central America, the Mayan and Mexican Indian samples overlap the birth locations for most of the LALES cohort.

### MEC Subjects

The Multiethnic Cohort (MEC) study is a prospective cohort of approximately 215,000 individuals from California and Hawaii [57]. This study was established between 1993-1996 and includes men and women primarily from five racial and ethnic populations in Hawaii and California (African Americans, European Americans, Latinos, Japanese Americans and Native Hawaiians). The USC MEP sample includes 355 individuals; 18 Chinese males from a prospective cohort from Shanghai, China [59], 17 females from the Singapore Health Study [60], 40 parents from 20 CEU trios from HapMap [69], and 280 MEC women without a history of cancer, namely, 70 Europeans, 70 African Americans, 70 Latinos from the Los Angeles area, 35 Japanese, and 35 Hawaiians. This multiethnic panel has been reported previously in de Bakker *et al*. [70] and Haiman *et al*. [69].

### Genotyping

The 538 LALES and 30 Native American subjects were genotyped using the Illumina GoldenGate platform for the 233 AIMs (USC Genomics Core Laboratory, Los Angeles, CA). The MEP panel samples were genotyped using the same platform (USC Genomics Core Laboratory, Los Angeles, CA). 176 SNPs out of 233 had genotype call rates > 0.98 and were chosen for the present analysis. Samples with an overall genotype call rate ≤ 0.8 were removed from analysis, resulting in a total of 500 LALES (250 cases, 250 controls) and 30 Native American individuals being included in the downstream analyses.

### Statistical Analysis

We employed a series of methods to evaluate the level of admixture among Latinos, to estimate the relative proportions of *AF*, *AS*, *EU*, and *NA *background in both LALES and MEC Latinos, and to assess the correlation of *NA *and *EU *ancestry with the LALES AMD case-control status. Ethnic proportions were inferred through the Markov chain Monte Carlo (MCMC) algorithm of Falush and Pritchard using the STRUCTURE 2.2 software package [71-73].

Assessment of Latino population admixture was performed using three different statistics: (1) the Pearson chi-square test to identify SNPs in Hardy Weinberg disequilibrium, (2) an overall assessment across all AIMs of the distribution of homozygous genotypes within each sampled population and also of that within US-born vs. non-US born Latinos, and (3) a measure for excess association between physically unlinked loci in LALES and in MEC Latinos.

### Estimation of Population Ancestry

The genetic make-up of LALES Latinos was inferred using the admixture modeling implemented in STRUCTURE 2.2 [71-73], and allowing for correlation between allele frequencies among populations. The ALPHA Dirichlet parameter for degree of admixture was inferred, starting at an initial value of 1.0 and a standard deviation of proposal for updating ALPHA of 0.025. We ran 45,000 burn-in repetitions and a further 50,000 iterations after the burn-in period. When using STRUCTURE, accurately deciding the number of clusters *K *that best describes a population's substructure is a rather difficult task [71-75]. Our solution was to focus on the value of *K *which not only captures most of the structure in a population, but also offers an experimentally relevant interpretation. We ran the analysis using different values of *K *and obtained the estimated log-likelihood of the data (*ln*Pr(X|*K*)) at each run. For each *K*-value three independent analyses were completed to ensure that *ln*Pr(X|*K*) estimates were consistent across runs. The average likelihood from the three independent runs is reported for each *K*, where the posterior probability of *K *can be computed as .

A second parameter of interest is the divergence in allele frequencies between the *K *clusters, traditionally referred to as Wright's *F*_*st *_measure [76]. The current STRUCTURE implementation reports *F*_*K*_, an analogue of *F*_*st*_, proposed by Falush *et al*. (2003) [73]. The *F*_*K*_-based model allows for variation in drift rates between populations, computing a different *F*_*K *_measure for each of the *K *populations rather than assessing an overall *F*_*st *_measure across all populations.

STRUCTURE analyses were performed first on the final set of 176 AIMs for the merged dataset of the LALES, *NA*, and USC MEP. These AIMs were selected from the high-density admixture map for disease gene discovery in African Americans (Smith *et *al., 2004); the STRUCTURE model integrates this information in estimating Latino ancestry. However, given the high heterogeneity among African populations (Tishkoff *et *al., 2009), we compared these estimates with those obtained from an additional analysis based on a subset of 111 SNPs for which 90 Yoruba Africans from the HapMap II database were also included in the ancestry model.

Since AIMs were selected for their lack of linkage with loci known to be associated with human diseases, the inclusion of cases would be unlikely to affect overall approximations. However, to avoid any potential biases we report the population structure results based only on the inclusion of LALES controls. In addition, as part of our continuing LALES Latino eye study we also completed a separate STRUCTURE analysis using only the 250 AMD cases. This additional step allowed us to further examine potential differences in ethnic background between AMD cases and controls by using the Wilcoxon signed test. Lastly, association between any of the AIMs and AMD status was tested using an additive genetic model. Allelic regression analysis was also conducted by including individual *EU *and *NA *ancestry estimates as model covariates for assessing the strength of association between any of the AIMs and AMD. Final p-values were corrected for multiple comparisons through Bonferroni adjustment at the 2.84*10^-4 ^(or 0.05/176) threshold.

### Identification of population structure and recent admixture

In a random-mating population we expect genotypes to be in Hardy-Weinberg equilibrium (HWE) [77]. Deviations from this equilibrium are typically thought to be due to population structure, selection or genotyping errors. For example, admixture will cause a modification of genotype frequencies in a population due to the influx of alleles from other populations [78]. Deviations due to selection are unlikely in the present context given that the AIMs were chosen to be optimal for distinguishing large scale population mixtures and for making precise ancestry estimates (Smith *et al*. 2004) [65]. Given this, we checked among Latinos for deviations from HWE in the set of 176 AIM SNPs using a Pearson's chi-square test with one degree of freedom. In addition, we tested for excess of homozygosity, a trademark of recent admixture. Choudhry and Siegmund implemented the *T *statistic measure for estimating the amount of deviation from HWE and the trend in homozygosity across all markers, where , *N *is the total number of individuals, *P*_*D *_and *P*_*d *_denote estimated allele frequencies, and *X*_*DD *_and *X*_*dd *_are the homozygote genotypic counts [36]. Under the assumption of HWE and based on the selection of randomly chosen genome-wide loci, a standard normal distribution is expected to fit the frequencies of the T-statistic [61], with heterozygote frequencies distributed towards the left, homozygote counts towards the right. The observed distribution of this *T*-statistic was contrasted between the LALES, MEC and Native American populations. We further searched for potential variation within Latinos themselves by evaluating regional specific homozygosity trends of individuals originating in different birthplace locations. A final analysis of population admixture was conducted by assessing the degree of allelic association between physically unlinked markers [16,61,79]. Any associations between AIM pairs from these SNP pairs would most likely be due to recent admixture or population substructure.

### Bootstrap Methods for Assessing the Effect of Sample Size on Population Structure Inference

An emerging concern when assessing ancestral proportions is the size of the genotyped samples within a given study. Two issues surface when inferring population structure: (1) the minimum sample size requirement for a given population, and (2) the difference in the size of the analyzed sub-populations. There is a danger that estimates of population ancestry might be influenced by the size of the (sub)population being analyzed. For example, it is plausible to imagine that it is easier to identify a population for which we have a large number of representatives than one with relatively few members. This is a particular concern in our study, given the discrepancies between sample sizes across ethnic groups, and this issue is not generally addressed in the literature. To guard against this issue we employed two commonly-used techniques for adjusting sample sizes. First, smaller samples were inflated by Boot-strapping (i.e. sampling at random with replacement) until they reached the LALES control sample size (n = 24 controls). Chinese and Japanese subjects were merged and categorized as 'Asians', while White and CEPH samples were grouped into a single 'European' population. We applied this scheme to inflate each of the following samples: 70 African Americans, 70 Latinos from LA (non-LALES), 35 Native Hawaiians, 70 Asians (35 Japanese, 18 Chinese from Shangai, and 17 Chinese from Singapore), and 110 Caucasians (40 CEPHs and 70 Europeans). Through a second approach we reduced the size of the LALES control cohort by selecting 70 individuals through random sampling without replacement. Unselected individuals were excluded from the subsequent STRUCTURE analysis. Each of the two schemes were repeated 100 times, and every resulting data-set was analyzed with STRUCTURE 2.2 under the K = 4 model parameterization used on the original data. We then reanalyzed the data to see if our earlier conclusions remained true.

## Authors' contributions

All authors read and approved the final manuscript. CJS contributed to the proposal of the study design, performed the statistical analysis, interpretation, and writing of the manuscript; PM contributed to the study design, statistical, and interpretative coordination of the project. He was involved in the revision and final approval of the manuscript; SA is co-investigator of the LALES cohort. He participated in the design, reviewing, and final approval of the manuscript; DVC contributed with the genotyping of the MEC AIMs data, advised on methods to be used, and gave critical reviews final approval of the manuscript; LLM participated in the genotyping of the MEC - Hawaiian data, methodology and review of the manuscript; CAH took part in the acquisition of the MEC AIMs data, advised on methodology and final manuscript review; RV is the main PI of the LALES cohort study and of the current population admixture project. He led and coordinated the acquisition of the LALES and Native American data, contributed to the merging of the LALES/Native American and MEC cohorts, proposed and guided this study, and gave interpretation, critical review, and final approval of the manuscript.

## Author's Information

CJS is a post-doctoral scholar in the department of Human Genetics and Neuroscience at the University of California - Los Angeles (UCLA); PM, DVC, and CAH are associate professors in the department of Biostatistics at USC, with research interests in the fields of Population Genetics, Statistical Genetics, and Biostatistics. DC and CAH are co-investigators of the MEC cohort; SA is dean of Biostatistics at USC, and co-investigator of the LALES study; LLM is professor of epidemiology at the University of Hawaii, Honolulu. He is principal investigator of the MEC cohort; RV is the director and PI of the LALES cohort study, and a Research to Prevent Blindness Sybil B. Harrington Scholar. Dr. Varma is director of the Glaucoma Service, Ocular Epidemiology Center and Clinical Trials, and professor in the department of Ophthalmology at USC.

## Supplementary Material

Additional file 1 **Additional Tables**. Table S1. Simulation summary statistics of ancestry clustering models. Table S2. Comparison of ancestry proportion medians (1^st ^: 3^rd ^quartile) among LALES Latinos by birth location and case-control status. Table S3. Range of ancestry proportion estimates (low - high) for LALES Latinos for random sets of 111 SNPs from the total 176 AIMs genotyped for the LALES, NA, and MEC cohorts. Table S4. Proportion of membership of each pre-defined population in each of the 5 clusters. Table S5. Ancestry informative markers with difference in allele frequency (*δ*) greater than 0.3 between Native American and European ancestry among Latinos. Table S6. Bootstrap simulation results for the increased and decreased sample size methods Click here for file

Additional file 2 **Figure S1**. Distribution of T-values for testing overall homozygosity and heterozygosity trends Click here for file

Additional file 3 **Figure S2**. Distribution of T-values for testing overall homozygosity and heterozygosity trends in LALES Latinos born within the US versus LALES Latinos born outside the US Click here for file

Additional file 4 **Figure S3**. Bootstrap re-sampling: distribution of European and Native American ancestry frequencies in LALES Latinos Click here for file
