# Supplementary material for: Variation in genetic admixture and population structure among Latinos: the Los Angeles Latino eye study (LALES)
Source: BMC Genet. 2009 Nov 10;10:71. doi: 10.1186/1471-2156-10-71 (PMC3087512; doi:10.1186/1471-2156-10-71)
Supplement: Additional file 1 — Additional Tables. Table S1. Simulation summary statistics of ancestry clustering models. Table S2. Comparison of ancestry proportion medians (1st : 3rd quartile) among LALES Latinos by birth location and case-control status. Table S3. Range of ancestry proportion estimates (low - high) for LALES Latinos for random sets of 111 SNPs from the total 176 AIMs genotyped for the LALES, NA, and MEC cohorts. Table S4. Proportion of membership of each pre-defined population in each of the 5 clusters. Table S5. Ancestry informative markers with difference in allele frequency (δ) greater than 0.3 between Native American and European ancestry among Latinos. Table S6. Bootstrap simulation results for the increased and decreased sample size methods [file 1471-2156-10-71-S1.doc]

**Supplementary Table 1**

**Simulation summary statistics of ancestry clustering models**

| **K** | **LnP(D)** | **Nucleotide Divergence Estimate** | | | | | | **Ancestry Proportion Estimates** | | | | | | | | | | | |
| --- | --- | --- | --- | --- | --- | --- | --- | --- | --- | --- | --- | --- | --- | --- | --- | --- | --- | --- | --- |
|  |  |  | | | | | | **LALES Latinos** | | | | | | **MEC Latinos** | | | | | |
|  |  | **F**_st_ **1** | **F**_st_ **2** | **F**_st_ **3** | **F**_st_ **4** | **F**_st_ **5** | **F**_st_ **6** | **C1** | **C2** | **C3** | **C4** | **C5** | **C6** | **C1** | **C2** | **C3** | **C4** | **C5** | **C6** |
| **2** | -122914.300 | 0.378 | 0.270 | - | - | - | - | 0.054 | 0.946 | - | - | - | - | 0.063 | 0.937 |  |  |  |  |
| **3** | -118001.900 | 0.269 | 0.314 | 0.529 | - | - | - | 0.056 | 0.387 | 0.557 | - | - | - | 0.070 | 0.445 | 0.484 | - | - | - |
| **4** | -116312.200 | 0.300 | 0.307 | 0.610 | 0.139 | - | - | 0.049 | 0.401 | 0.452 | 0.098 | - | - | 0.059 | 0.453 | 0.373 | 0.116 | - | - |
| **5** | -116186.100 | 0.202 | 0.168 | 0.004 | 0.275 | 0.623 | - | 0.019 | 0.137 | 0.173 | 0.041 | 0.630 | - | 0.037 | 0.253 | 0.176 | 0.058 | 0.476 | - |

Note.

For each *K*, the results are based on the average of 3 independent MCMC runs, each of the 3 iterations having been initialized through a different seed.

LnP(D) = Log Likelihood Probability of the Data

F_st_ = Nucleotide distance or divergence between predefined clusters

C = Inferred Cluster

**Supplementary Table 2**

**Comparison of ancestry proportion medians (1^st^ : 3^rd^ quartile) among LALES Latinos by birth location and case-control status**

| **Birth Origin** | **African American** | |  | **European** | |  | **Asian** | |  | **Native American** | |
| --- | --- | --- | --- | --- | --- | --- | --- | --- | --- | --- | --- |
|  | Cases | Controls |  | Cases | Controls |  | Cases | Controls |  | Cases | Controls |
| **El Salvador** | 0.057 (0.029:0.095) | 0.034 (0.017:0.067) |  | 0.358 (0.282:0.455) | 0.272 (0.187:0.376) |  | 0.053 (0.026:0.210) | 0.058 (0.0.03:0.125) |  | 0.483 (0.380:0.511) | 0.581 (0.405:0.653) |
| **Guatemala** | 0.035 (0.018:0.093) | 0.071 (0.018:0.089) |  | 0.283 (0.229:0.316) | 0.366 (0.289:0.444) |  | 0.022 (0.018:0.102) | 0.034 (0.022:0.043) |  | 0.628 (0.506:674) | 0.576 (0.515:0.609) |
| **Mexico** | 0.031 (0.016:0.057) | 0.029 (0.013:0.059) |  | 0.404 (0.261:0.519) | 0.408 (0.302:0.503) |  | 0.059 (0.029:0.124) | 0.068 (0.038:0.131) |  | 0.460 (0.334:0.606) | 0.452 (0.345:0.547) |
| **Other** | 0.058 (0.042:0.119) | 0.0.051 (0.031:0.083) |  | 0.391 (0.285:0.626) | 0.419 (0.370:0.501) |  | 0.047 (0.024:0.079) | 0.054 (0.042:0.092) |  | 0.337 (0.115:0.555) | 0.462 (0.366:0.511) |
| **USA** | 0.023 (0.013:0.047) | 0.038 (0.016:0.073) |  | 0.422 (0.336:0.0514) | 0.378 (0.289:0.511) |  | 0.055 (0.037:0.090) | 0.069 (0.034:0.12) |  | 0.457 (0.368:0.529) | 0.457 (0.331:0.620) |
| **ALL** | 0.031 (0.016:0.058) | 0.032 (0.013:0.064) |  | 0.404 (0.284:0.513) | 0.371 (0.290:0.502) |  | 0.057 (0.029:0.118) | 0.066 (0.036:0.126) |  | 0.466 (0.345:0.594) | 0.460 (0.345:0.576) |

**Supplementary Table 3**

**Range of ancestry proportion estimates (low – high) for LALES Latinos for random sets of 111 SNPs from the total 176 AIMs genotyped for the LALES, NA, and MEC cohorts.**

| **Source Populations** | **Regional LALES Estimates** | ***NA*** | ***EU*** | ***AF*** | ***AS*** |
| --- | --- | --- | --- | --- | --- |
| **AA, AS, NA, EU** | All | 0.422 - 0.514 | 0.321 - 0.379 | 0.047 - 0.058 | 0.110 - 0.141 |
|  | El Salvador/Guatemala | 0.484 - 0.557 | 0.228 - 0.259 | 0.081 - 0.095 | 0.121 - 0.163 |
|  | Mexico | 0.444 - 0.540 | 0.309 - 0.374 | 0.039 - 0.050 | 0.104 - 0.136 |
|  | USA | 0.320 - 0.457 | 0.378 - 0.458 | 0.034 - 0.051 | 0.127 - 0.171 |

Note.

LALES = Los Angeles Latino Eye Study

*AA* – African American; *AF* – African; *AS* – Asian; *EU* – European; *NA* – Native American

**Supplementary Table 4**

**Proportion of membership of each pre-defined population in each of the 5 clusters.**

| **Population** | ***AF*** | ***EU*** | ***AS*** | ***NA*** | **5^th^ CLUSTER** |
| --- | --- | --- | --- | --- | --- |
| MEC African American | 0.736 | 0.138 | 0.042 | 0.025 | 0.059 |
| MEC Native Hawaiian | 0.023 | 0.292 | 0.589 | 0.018 | 0.079 |
| MEC Japanese | 0.006 | 0.020 | 0.92 | 0.033 | 0.021 |
| Chinese - Shanghai | 0.006 | 0.008 | 0.945 | 0.027 | 0.014 |
| Chinese - Singapore | 0.007 | 0.009 | 0.956 | 0.014 | 0.013 |
| MEC European | 0.005 | 0.959 | 0.011 | 0.009 | 0.016 |
| CEPH | 0.009 | 0.893 | 0.036 | 0.013 | 0.049 |
| Native American | 0.003 | 0.008 | 0.022 | 0.947 | 0.020 |
| MEC Latinos | 0.037 | 0.253 | 0.058 | 0.176 | 0.476 |
| LALES Latinos | 0.019 | 0.137 | 0.041 | 0.173 | 0.630 |

Note.

Estimates for the LALES Latino population are based on the LALES controls.

African – *AF*; Asian – *AS*; European – *EU*; Native American – *NA*

LALES = Los Angeles Latino Eye Study

MEC = Multi-Ethnic Cohort

**Supplementary Table 5**

**Ancestry informative markers with difference in allele frequency (*δ*) greater than 0.3 between Native American and European ancestry among Latinos.**

| **Marker Information** | | | |  | **MAF** |  | **Allele Frequency** | | | |  | **δ** | | |
| --- | --- | --- | --- | --- | --- | --- | --- | --- | --- | --- | --- | --- | --- | --- |
| **Locus** | Allele | CHR | Position |  | Latinos |  | *NA* | *EU* | *AS* | *AA* |  | *NA*/*EU* | *NA*/*AS* | *NA*/*AA* |
| **rs6662385** | T | 1 | 72,946,163 |  | 0.35 |  | 0.66 | 0.20 | 0.77 | 0.01 |  | 0.46 | 0.11 | 0.65 |
|  | C | 1 | 72,946,163 |  | 0.35 |  | 0.34 | 0.80 | 0.23 | 0.99 |  | 0.46 | 0.11 | 0.65 |
| **CV11745078** | G | 2 | 177,911,950 |  | 0.28 |  | 0.04 | 0.37 | 0.48 | 0.98 |  | 0.34 | 0.45 | 0.94 |
|  | A | 2 | 177,911,950 |  | 0.28 |  | 0.96 | 0.63 | 0.52 | 0.02 |  | 0.34 | 0.45 | 0.94 |
| **rs1275988** | C | 2 | 26,767,868 |  | 0.31 |  | 0.10 | 0.87 | 0.36 | 0.91 |  | 0.77 | 0.26 | 0.81 |
|  | T | 2 | 26,767,868 |  | 0.31 |  | 0.90 | 0.13 | 0.64 | 0.09 |  | 0.77 | 0.26 | 0.81 |
| **rs2060447** | C | 2 | 163,308,799 |  | 0.23 |  | 0.89 | 0.17 | 0.86 | 0.02 |  | 0.72 | 0.03 | 0.87 |
|  | T | 2 | 163,308,799 |  | 0.23 |  | 0.11 | 0.83 | 0.14 | 0.98 |  | 0.72 | 0.03 | 0.87 |
| **rs2625051** | C | 2 | 131,229,320 |  | 0.17 |  | 0.90 | 0.56 | 0.87 | 0.04 |  | 0.34 | 0.03 | 0.87 |
|  | T | 2 | 131,229,320 |  | 0.17 |  | 0.10 | 0.44 | 0.13 | 0.97 |  | 0.34 | 0.03 | 0.87 |
| **CV74522** | T | 3 | 115,646,846 |  | 0.45 |  | 0.21 | 0.50 | 0.74 | 0.19 |  | 0.30 | 0.53 | 0.02 |
|  | C | 3 | 115,646,846 |  | 0.45 |  | 0.79 | 0.50 | 0.26 | 0.81 |  | 0.30 | 0.53 | 0.02 |
| **rs3796384** | C | 3 | 64,501,757 |  | 0.45 |  | 0.10 | 0.41 | 0.86 | 0.24 |  | 0.30 | 0.75 | 0.14 |
|  | G | 3 | 64,501,757 |  | 0.45 |  | 0.90 | 0.59 | 0.15 | 0.76 |  | 0.30 | 0.75 | 0.14 |
| **rs7611703** | T | 3 | 2,749,996 |  | 0.47 |  | 0.43 | 0.72 | 0.70 | 0.10 |  | 0.30 | 0.27 | 0.33 |
|  | C | 3 | 2,749,996 |  | 0.47 |  | 0.57 | 0.28 | 0.31 | 0.90 |  | 0.30 | 0.27 | 0.33 |
| **rs1921877** | C | 4 | 85,181,677 |  | 0.48 |  | 0.28 | 0.61 | 0.73 | 0.15 |  | 0.34 | 0.46 | 0.13 |
|  | T | 4 | 85,181,677 |  | 0.48 |  | 0.72 | 0.39 | 0.27 | 0.85 |  | 0.34 | 0.46 | 0.13 |
| **rs262838** | G | 5 | 169,107,272 |  | 0.47 |  | 0.76 | 0.29 | 0.14 | 0.72 |  | 0.47 | 0.63 | 0.04 |
|  | A | 5 | 169,107,272 |  | 0.47 |  | 0.24 | 0.71 | 0.86 | 0.28 |  | 0.47 | 0.63 | 0.04 |
| **rs4702813** | G | 5 | 11,795,668 |  | 0.32 |  | 0.82 | 0.04 | 0.71 | 0.01 |  | 0.78 | 0.11 | 0.82 |
|  | A | 5 | 11,795,668 |  | 0.32 |  | 0.18 | 0.96 | 0.29 | 0.99 |  | 0.78 | 0.11 | 0.82 |
| **rs874973** | A | 5 | 72,773,651 |  | 0.13 |  | 0.89 | 0.58 | 0.94 | 0.06 |  | 0.31 | 0.06 | 0.82 |
|  | G | 5 | 72,773,651 |  | 0.13 |  | 0.11 | 0.42 | 0.06 | 0.94 |  | 0.31 | 0.06 | 0.82 |
| **rs900379** | T | 5 | 44,405,413 |  | 0.41 |  | 0.18 | 0.54 | 0.66 | 0.18 |  | 0.36 | 0.48 | 0.01 |
|  | C | 5 | 44,405,413 |  | 0.41 |  | 0.82 | 0.46 | 0.34 | 0.82 |  | 0.36 | 0.48 | 0.01 |
| **CV11635757** | A | 6 | 121,696,295 |  | 0.46 |  | 0.83 | 0.25 | 0.18 | 0.82 |  | 0.58 | 0.65 | 0.01 |
|  | G | 6 | 121,696,295 |  | 0.46 |  | 0.17 | 0.75 | 0.82 | 0.18 |  | 0.58 | 0.65 | 0.01 |
| **rs1480642** | T | 6 | 136,541,221 |  | 0.26 |  | 0.45 | 0.10 | 0.00 | 0.85 |  | 0.35 | 0.45 | 0.40 |
|  | C | 6 | 136,541,221 |  | 0.26 |  | 0.55 | 0.90 | 1.00 | 0.15 |  | 0.35 | 0.45 | 0.40 |
| **rs222541** | G | 6 | 95,287,884 |  | 0.26 |  | 0.91 | 0.50 | 0.70 | 0.02 |  | 0.41 | 0.21 | 0.89 |
|  | C | 6 | 95,287,884 |  | 0.26 |  | 0.09 | 0.50 | 0.30 | 0.98 |  | 0.41 | 0.21 | 0.89 |
| **rs606548** | C | 6 | 53,510,638 |  | 0.41 |  | 0.00 | 0.73 | 0.61 | 0.61 |  | 0.73 | 0.61 | 0.60 |
|  | T | 6 | 53,510,638 |  | 0.41 |  | 1.00 | 0.27 | 0.39 | 0.39 |  | 0.73 | 0.61 | 0.60 |
| **rs1031402** | A | 8 | 109,158,764 |  | 0.27 |  | 0.93 | 0.56 | 0.68 | 0.01 |  | 0.37 | 0.25 | 0.93 |
|  | G | 8 | 109,158,764 |  | 0.27 |  | 0.07 | 0.44 | 0.32 | 0.99 |  | 0.37 | 0.25 | 0.93 |
| **rs2045638** | A | 8 | 2,963,312 |  | 0.16 |  | 0.93 | 0.58 | 0.83 | 0.01 |  | 0.35 | 0.10 | 0.92 |
|  | G | 8 | 2,963,312 |  | 0.16 |  | 0.07 | 0.43 | 0.17 | 0.99 |  | 0.35 | 0.10 | 0.92 |
| **rs2076974** | C | 10 | 111,810,301 |  | 0.15 |  | 0.99 | 0.57 | 0.85 | 0.14 |  | 0.42 | 0.14 | 0.85 |
|  | T | 10 | 111,810,301 |  | 0.15 |  | 0.01 | 0.43 | 0.15 | 0.86 |  | 0.42 | 0.14 | 0.85 |
| **rs959354** | C | 11 | 129,514,791 |  | 0.09 |  | 0.01 | 0.39 | 0.08 | 0.88 |  | 0.39 | 0.07 | 0.88 |
|  | T | 11 | 129,514,791 |  | 0.09 |  | 0.99 | 0.61 | 0.92 | 0.12 |  | 0.39 | 0.07 | 0.88 |
| **CV11287912** | C | 12 | 54,471,828 |  | 0.34 |  | 0.37 | 0.87 | 0.96 | 0.24 |  | 0.50 | 0.58 | 0.14 |
|  | A | 12 | 54,471,828 |  | 0.34 |  | 0.63 | 0.13 | 0.05 | 0.76 |  | 0.50 | 0.58 | 0.14 |
| **rs2293048** | T | 12 | 116,149,208 |  | 0.42 |  | 0.68 | 0.15 | 0.12 | 0.57 |  | 0.52 | 0.56 | 0.10 |
|  | C | 12 | 116,149,208 |  | 0.42 |  | 0.32 | 0.85 | 0.88 | 0.43 |  | 0.52 | 0.56 | 0.10 |
| **rs4766807** | A | 12 | 115,785,834 |  | 0.30 |  | 0.92 | 0.45 | 0.62 | 0.01 |  | 0.47 | 0.30 | 0.91 |
|  | T | 12 | 115,785,834 |  | 0.30 |  | 0.08 | 0.55 | 0.38 | 1.00 |  | 0.47 | 0.30 | 0.91 |
| **rs7995033** | T | 13 | 24,729,888 |  | 0.47 |  | 0.76 | 0.41 | 0.13 | 0.96 |  | 0.34 | 0.63 | 0.20 |
|  | C | 13 | 24,729,888 |  | 0.47 |  | 0.25 | 0.59 | 0.87 | 0.04 |  | 0.34 | 0.63 | 0.20 |
| **CV1436495** | C | 14 | 20,994,513 |  | 0.30 |  | 0.10 | 0.40 | 0.51 | 0.00 |  | 0.30 | 0.41 | 0.09 |
|  | G | 14 | 20,994,513 |  | 0.30 |  | 0.90 | 0.61 | 0.49 | 1.00 |  | 0.30 | 0.41 | 0.09 |
| **rs6495569** | A | 15 | 79,447,292 |  | 0.14 |  | 0.99 | 0.69 | 0.81 | 0.17 |  | 0.31 | 0.18 | 0.82 |
|  | G | 15 | 79,447,292 |  | 0.14 |  | 0.01 | 0.31 | 0.19 | 0.83 |  | 0.31 | 0.18 | 0.82 |
| **rs2217271** | C | 16 | 12,611,981 |  | 0.26 |  | 0.88 | 0.50 | 0.70 | 0.24 |  | 0.38 | 0.18 | 0.64 |
|  | A | 16 | 12,611,981 |  | 0.26 |  | 0.13 | 0.50 | 0.30 | 0.76 |  | 0.38 | 0.18 | 0.64 |
| **rs878522** | G | 20 | 45,050,583 |  | 0.28 |  | 0.98 | 0.65 | 0.37 | 0.98 |  | 0.34 | 0.62 | 0.00 |
|  | A | 20 | 45,050,583 |  | 0.28 |  | 0.02 | 0.35 | 0.63 | 0.02 |  | 0.34 | 0.62 | 0.00 |
| **rs727563** | T | 22 | 40,197,323 |  | 0.40 |  | 0.03 | 0.60 | 0.79 | 0.24 |  | 0.57 | 0.76 | 0.21 |
|  | C | 22 | 40,197,323 |  | 0.40 |  | 0.98 | 0.40 | 0.21 | 0.76 |  | 0.57 | 0.76 | 0.21 |

Note.

SNP ID = Marker Name

CHR = Chromosome

δ = Allele frequency difference

**Supplementary Table 6**

**Bootstrap simulation results for the increased and decreased sample size methods.**

| **Bootstrap**  **Method** | **Statistics** | **EU**  **Ancestry** | ***NA***  **Ancestry** |
| --- | --- | --- | --- |
| Increased Sample Size | Mean | 0.44 | 0.42 |
|  | Median | 0.44 | 0.42 |
|  | Variance | 4.38E-05 | 5.50E-05 |
|  | S.D. | 6.61E-03 | 7.42E-03 |
|  | Minimum | 0.42 | 0.39 |
|  | Maximum | 0.47 | 0.44 |
| Decreased Sample Size | Mean | 0.42 | 0.45 |
|  | Median | 0.42 | 0.45 |
|  | Variance | 2.54E-04 | 3.43E-04 |
|  | S.D. | 0.02 | 0.02 |
|  | Minimum | 0.38 | 0.39 |
|  | Maximum | 0.46 | 0.48 |

Note.

Original sample analysis gave estimates for LALES Latinos of 45.2% Native American and 40.1 % European ancestry

S.D. = Standard Deviation

*EU* = European; *NA* = Native American
