# Supplementary material for: Variation in genetic admixture and population structure among Latinos: the Los Angeles Latino eye study (LALES)
Source: BMC Genet. 2009 Nov 10;10:71. doi: 10.1186/1471-2156-10-71 (PMC3087512; doi:10.1186/1471-2156-10-71)
Supplement: Additional file 2 — Figure S1. Distribution of T-values for testing overall homozygosity and heterozygosity trends [file 1471-2156-10-71-S2.pdf]

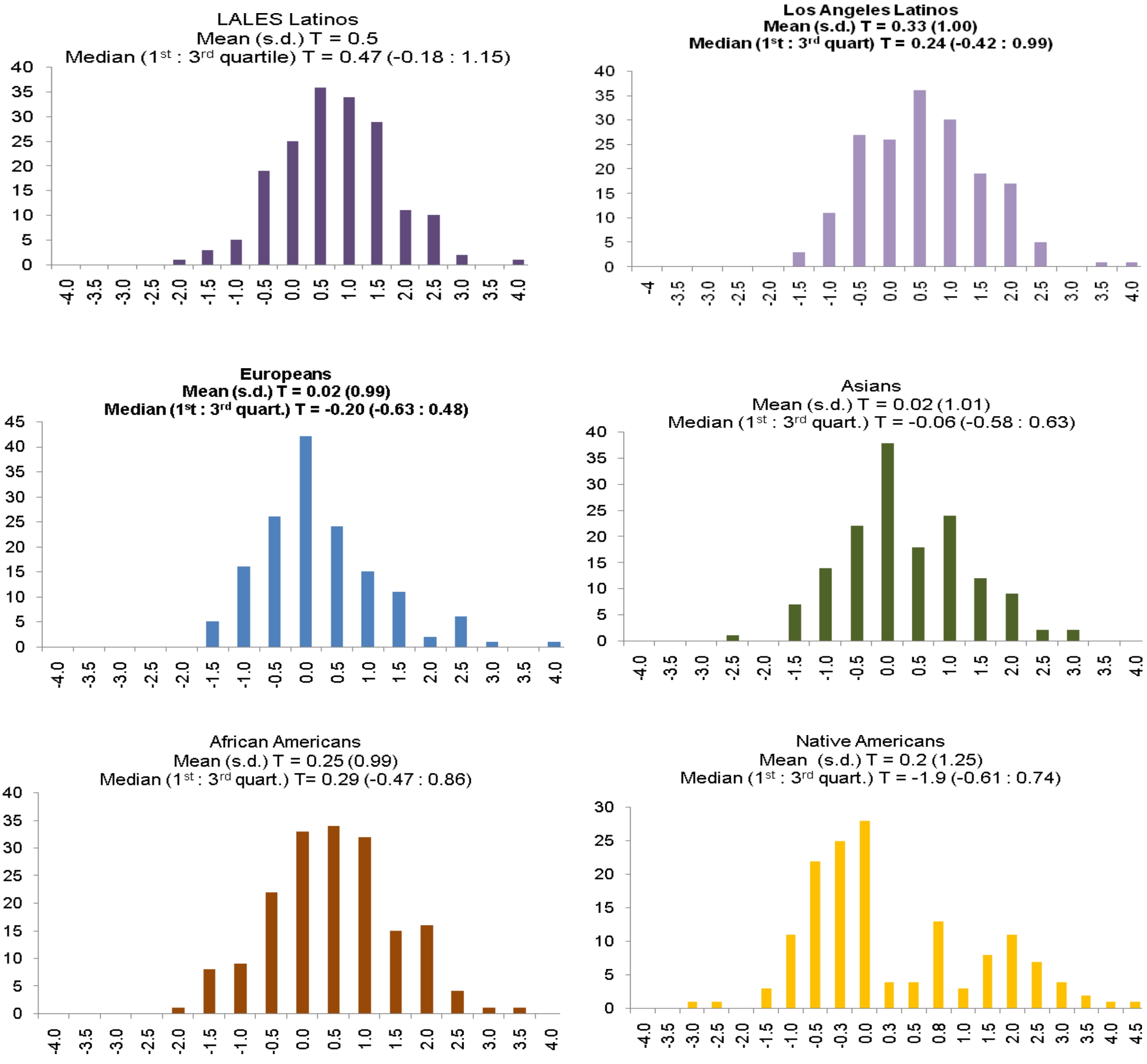
LALES Latinos
Mean (s.d.) T = 0.5
Median (1st : 3rd quartile) T = 0.47 (-0.18 : 1.15)
Los Angeles Latinos
Mean (s.d.) T = 0.33 (1.00)
Median (1st : 3rd quart) T = 0.24 (-0.42 : 0.99)
Europeans
Mean (s.d.) T = 0.02 (0.99)
Median (1st : 3rd quart.) T = -0.20 (-0.63 : 0.48)
Asians
Mean (s.d.) T = 0.02 (1.01)
Median (1st : 3rd quart.) T = -0.06 (-0.58 : 0.63)
African Americans
Mean (s.d.) T = 0.25 (0.99)
Median (1st : 3rd quart.) T= 0.29 (-0.47 : 0.86)
Native Americans
Mean (s.d.) T = 0.2 (1.25)
Median (1st : 3rd quart.) T = -1.9 (-0.61 : 0.74)
