# Supplementary material for: Variation in genetic admixture and population structure among Latinos: the Los Angeles Latino eye study (LALES)
Source: BMC Genet. 2009 Nov 10;10:71. doi: 10.1186/1471-2156-10-71 (PMC3087512; doi:10.1186/1471-2156-10-71)
Supplement: Additional file 3 — Figure S2. Distribution of T-values for testing overall homozygosity and heterozygosity trends in LALES Latinos born within the US versus LALES Latinos born outside the US [file 1471-2156-10-71-S3.pdf]

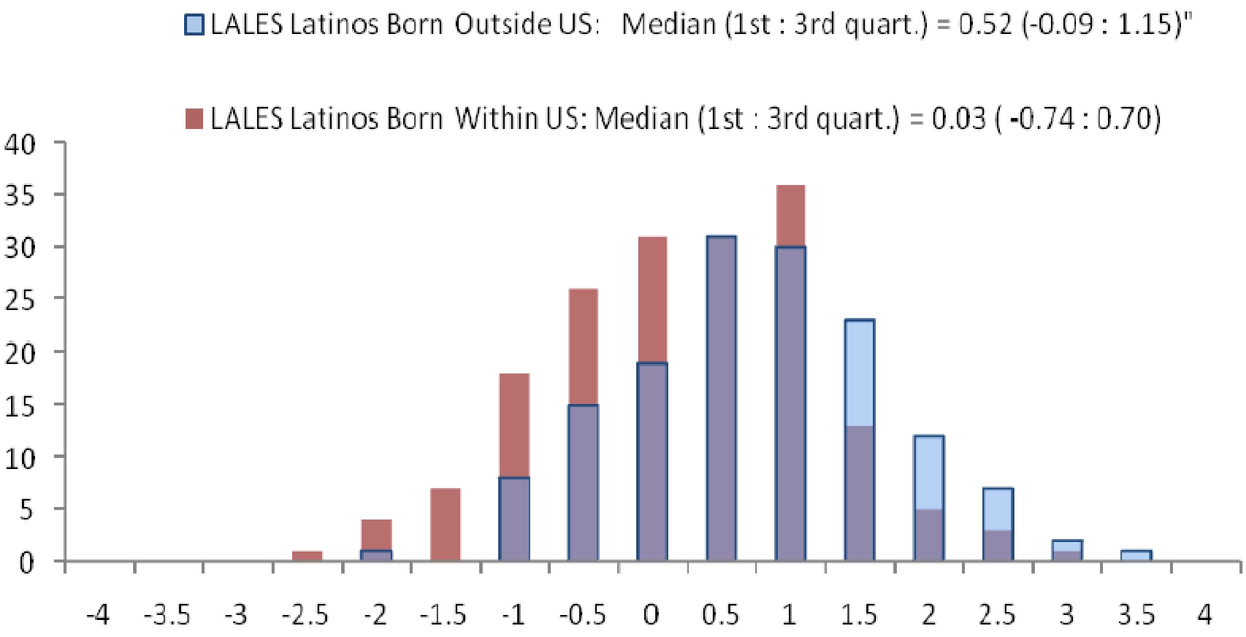
LALES Latinos Born Outside US: Median (1st : 3rd quart.) = 0.52 (-0.09 : 1.15)"
LALES Latinos Born Within US: Median (1st : 3rd quart.) = 0.03 (-0.74 : 0.70)
0 5 10 15 20 25 30 35 40
-4 -3.5 -3 -2.5 -2 -1.5 -1 -0.5 0 0.5 1 1.5 2 2.5 3 3.5 4

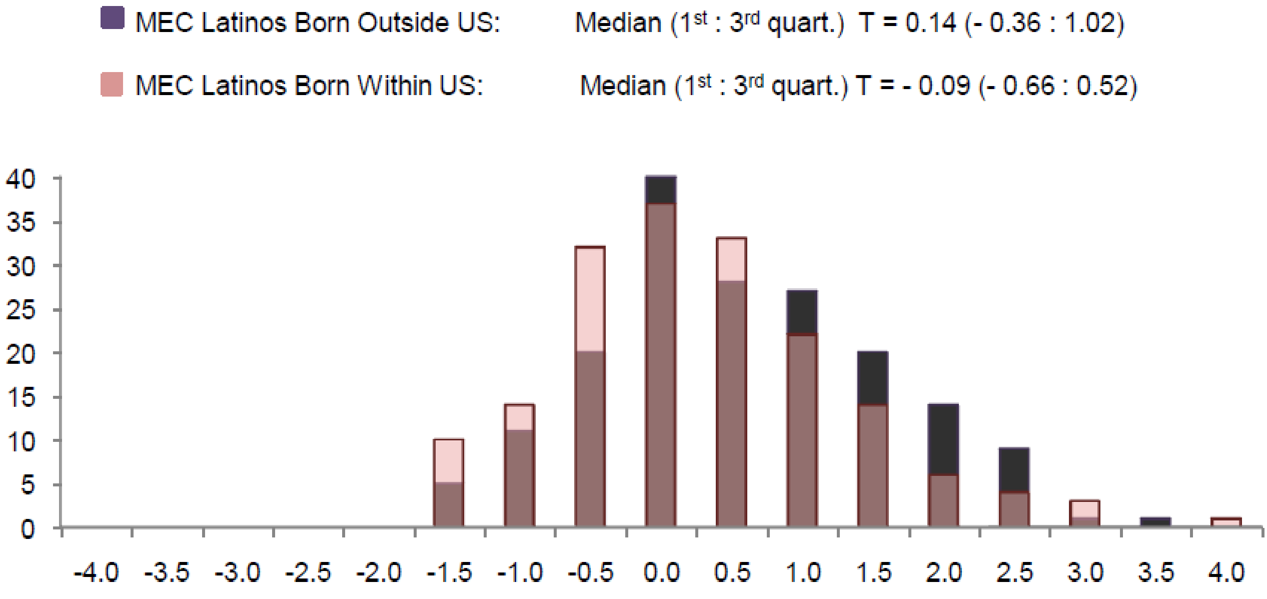
MEC Latinos Born Outside US: Median (1st : 3rd quart.) T = 0.14 (- 0.36 : 1.02)
MEC Latinos Born Within US: Median (1st : 3rd quart.) T = - 0.09 (- 0.66 : 0.52)
0 5 10 15 20 25 30 35 40
-4.0 -3.5 -3.0 -2.5 -2.0 -1.5 -1.0 -0.5 0.0 0.5 1.0 1.5 2.0 2.5 3.0 3.5 4.0
