# Supplementary figures and images for: Variation in genetic admixture and population structure among Latinos: the Los Angeles Latino eye study (LALES)
Source: BMC Genet. 2009 Nov 10;10:71. doi: 10.1186/1471-2156-10-71 (PMC3087512; doi:10.1186/1471-2156-10-71)

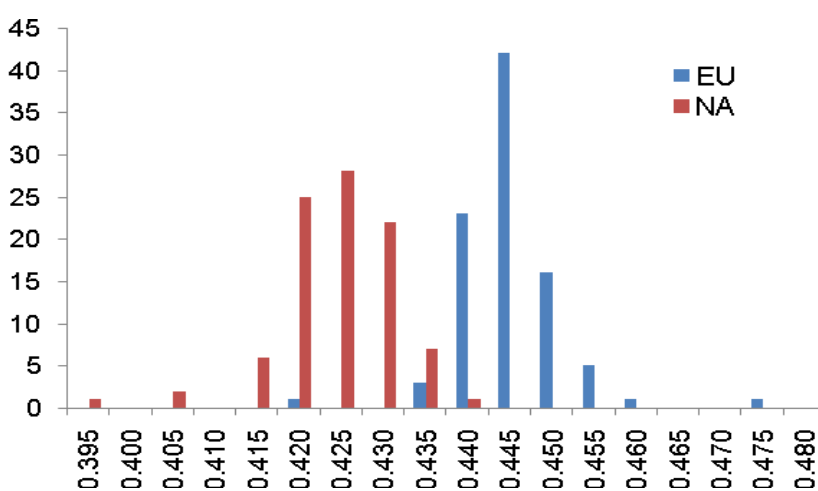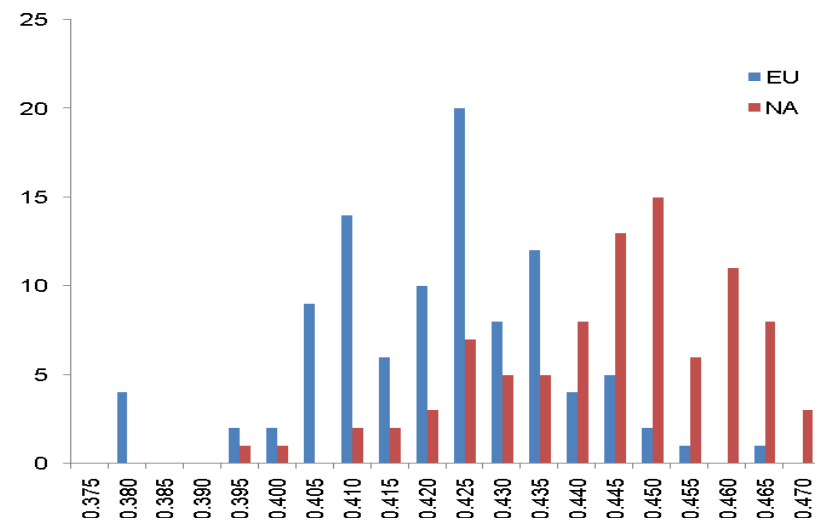

Supplement: Additional file 4 — Figure S3. Bootstrap re-sampling: distribution of European and Native American ancestry frequencies in LALES Latinos [file 1471-2156-10-71-S4.pdf]
